# Treatment of Binge-Eating Disorder Across the Lifespan: An Updated Review of the Literature and Considerations for Future Research

**DOI:** 10.1007/s13679-024-00553-4

**Published:** 2024-02-16

**Authors:** Kathryn E. Smith, Andrea B. Goldschmidt

**Affiliations:** 1 Department of Psychiatry and Behavioral Sciences, University of Southern California, 2250 Alcazar St #2200, Los Angeles, CA 90033 USA; 2 Department of Psychiatry, University of Pittsburgh School of Medicine, Pittsburgh, PA USA

**Keywords:** Binge-eating disorder, Treatment, Psychotherapy, Pharmacotherapy, Review, Efficacy

## Abstract

**Purpose of Review:**

The present review describes the recent literature on treatment for binge-eating disorder (BED) in adults and youth, with a particular focus on research gaps, emerging treatments, and future research directions.

**Recent Findings:**

Evidence supports the efficacy of several treatment modalities in adults, including self-help treatment, clinician-led psychotherapy, and pharmacotherapy; the largest effect sizes have been found for psychotherapies, most of which were cognitive-behavioral in orientation. Adapted psychotherapies for youth also show promise but lack a robust body of evidence. Predictors, moderators, and mediators of treatment outcome remain poorly understood; individuals with BED continue to experience significant barriers to treatment; and research is needed to address suboptimal treatment response. Recent work has highlighted the potential of adaptive interventions and investigation of novel mechanisms to address these gaps.

**Summary:**

Research on BED treatment continues to grow, though critical questions must be answered to improve treatment efficacy across the lifespan.

## Introduction

Binge-eating disorder (BED) is characterized by recurrent episodes of binge eating (i.e., consuming an objectively large amount of food while experiencing a sense of loss of control over eating) in the absence of regular use of inappropriate compensatory behaviors intended to influence body weight [1]. Recent point prevalence estimates indicate BED affects ~ 1–4% of adults [2] and ~ 1% of youth [3] and is strongly associated with elevated body mass index (BMI; kg/m^2^ [4]), excess weight gain [5–7], and physical [8, 9] and psychiatric comorbidities [10], including components of metabolic syndrome, gastrointestinal symptoms, mood and anxiety disorders, and sleep- and pain-related conditions. As such, BED presents significant burden to the individual and to society, costing consumers nearly $20 billion each year in direct healthcare expenditures, lost wages, and decreased productivity [11]. There are several efficacious interventions for BED in adults, including psychological, behavioral, pharmacological, and surgical treatment options [12], with fewer well-established (but nevertheless promising) treatment options for children and adolescents [13]. The purpose of this article is to provide an updated review on the treatment of BED across the lifespan, with special attention to new and emerging interventions and areas for future research.

## Adult Treatment Approaches

### Psychological Interventions

Several empirically supported treatment frameworks are available for adults with BED. First-line interventions for those with mild-to-moderate symptom severity include cognitive-behavioral therapy (CBT), which focuses primarily on establishing regular eating patterns and addressing maladaptive cognitions related to body shape and weight, the putative maintaining factors of binge-eating behaviors [14], and interpersonal psychotherapy (IPT), which focuses on improving problematic social factors that drive negative affect and subsequent binge eating. Emerging research also supports the efficacy of integrative cognitive-affective therapy (ICAT), which targets momentary cognitive, behavioral, and emotional antecedents to binge eating, and adapted third-wave interventions such as dialectical behavior therapy (DBT), acceptance and commitment therapy (ACT), mindfulness-based interventions, and compassion-focused therapy [15•,^16^–^18^]. While these interventions are typically delivered in individual or group-based formats, several approaches have been adapted into self-help and/or digital formats to improve access and reduce costs.

To date, CBT has been the most commonly studied intervention for BED with the largest body of supporting evidence, and as such, it forms the foundation for most currently available self-help interventions. The efficacy of self-help treatments has been supported by randomized controlled trials (RCTs) showing reductions in binge-eating symptoms and eating-related psychopathology compared to wait-list controls, with effects maintained up to 12-months post-treatment [15•, 19•]. Similarly, with respect to clinician-led psychotherapy, recent meta-analyses showed that compared to inactive control conditions, psychotherapy (consisting of mostly CBT) showed large effect sizes in reducing binge-eating episodes and eating disorder psychopathology at end-of-treatment and through 12-months post-treatment [15•, 18]. Notably, thus far, there remains limited evidence supporting the superiority of CBT compared to other active psychotherapies or treatment modalities [15•, 16]. However, some evidence suggests that clinician-led CBT may produce more rapid change compared to IPT and self-help treatment, and was associated with lower drop-out compared to self-help treatment; additionally, CBT was more efficacious in reducing binge eating compared to behavioral weight loss interventions [15•, 17, 20].

### Pharmacological and Medical Interventions

For adults with moderate-to-severe BED symptoms, evidence also supports the efficacy of pharmacotherapy alone or in conjunction with psychotherapy [15•, 21]. Although no drug has been developed specifically for binge eating or BED, several psychotropic medications targeting other conditions have shown to also have beneficial effects on binge eating and have been re-purposed for BED treatment. Currently, the stimulant medication lisdexamfetamine (LDX) is the only FDA-approved medication in the United States for BED treatment, though antidepressants (particularly selective serotonin reuptake inhibitors) and antiepileptics (particularly topiramate) have also shown modest efficacy in reducing binge eating in BED [21]. In addition, although bariatric surgery is not considered a primary intervention for BED, most studies have found that bariatric surgery is related to initial improvements in binge eating and BED, which are usually shown through two years post-surgery [22]. However, while binge eating improves in the short-term after surgery, this behavior may rebound over time, and the course of postoperative binge eating becomes more variable over longer term follow-ups [22]. The initial declines in binge eating could be due in part to anatomical changes that limit the capacity to objectively overeat following surgery, and some research suggests other problematic eating behaviors may emerge or worsen during this time, such as loss of control eating and grazing (i.e., repetitive, unplanned consumption of small quantities of food over time) [22].

### Limitations and Research Gaps

Despite the variety of available evidence-based interventions, there remain critical limitations and unanswered questions in the treatment of BED in adults. Access to and affordability of treatment remains a significant barrier across eating disorder diagnoses [23], and the majority of individuals with eating disorders, including those with BED, do not seek out and/or engage in treatment [24]. Treatment is often delayed due to under-detection and under-diagnosis of BED by healthcare professionals, particularly in primary care settings where individuals are most likely to first seek help [24, 25]. Additionally, those with BED may not identify their symptoms as problematic due to limited mental health literacy about eating disorders, and individuals are more likely to seek help for physical rather than mental health complaints [26, 27]. These barriers may be exacerbated in BED due to stereotypes that eating disorders are limited to those who are underweight or engage in restriction or purging behaviors, as well as experiences of stigma [28, 29]. For instance, compared to individuals with anorexia nervosa or bulimia nervosa, those with BED are more likely to be the target of stigmatizing attributions such as lack of self-control, personal responsibility, and blameworthiness [30]. These beliefs may also be present among family, friends, and healthcare professionals of those with BED, further hindering the recognition of BED symptoms and likelihood that affected individuals will seek out treatment.

Even when individuals with BED seek out help, most do not receive specialized eating disorder treatment [24]. And although more efficacious than inactive control groups, the outcomes of existing specialized treatments are modest, with about half of individuals with BED achieving abstinence from binge eating at end-of-treatment through 12-months post-treatment [19•]. Notably, despite the effects on binge eating, psychotherapies for BED do not lead to consistent changes in body weight [15•, 19•]. A recent comprehensive meta-analysis of BED treatment outcomes across 81 RCTs (including self-help, psychotherapy, and pharmacotherapy interventions) also demonstrated high heterogeneity across studies, low methodological quality, and a lack of long-term follow-up data examining durability of effects [15•]. In addition, there were few head-to-head comparisons between BED treatments, limiting understanding of comparative efficacy of interventions [15•].

Finally, little is known regarding predictors, moderators, and mediators of BED treatment outcomes, which are crucial in helping to identify which treatments work best for whom, as well as pinpointing underlying mechanisms of action. Consideration of heterogeneity in treatment response is imperative given earlier research showing individuals with less severe BED symptoms responded to placebo [31], suggesting that a subset of individuals could benefit from lower intensity treatments and may experience more a transient course of illness. With respect to predictors of treatment outcomes, across treatment modalities, meta-analytic results identified some patient characteristics (i.e., lower age and BMI, female sex, and higher baseline binge-eating episodes) as non-specific predictors that related to greater reduction in binge-eating episodes [15•]. Thus far, early behavior change (characterized by a 65–70% reduction in binge eating by week 4 of treatment) has been the only consistent predictor of CBT outcome [32]. With respect to moderators, patient demographics and psychiatric comorbidities have not consistently predicted response to CBT compared to other treatments for BED, and inconsistent findings have emerged regarding overvaluation of shape and weight as a differential predictor of CBT outcomes compared to other psychotherapies [32]. With respect to mindfulness- and acceptance-based treatments interventions for BED, a recent review showed that few studies assessed moderators of outcomes [33]. Regarding mediators, research has been limited and inconsistent across treatment modalities. A recent study demonstrated support for mechanisms implicated by interpersonal theory, showing that the relationship between decreases in interpersonal problems and binge-eating reduction was mediated by improvement in negative affect in both CBT and IPT [34]. In addition, the review of mindfulness- and acceptance-based treatments suggested that pre- to post-treatment improvements in theoretically consistent mechanisms (e.g., psychological flexibility, emotion regulation) were associated with better outcomes, though rigorous statistical tests of mediation were lacking [33].

## Child and Adolescent Treatment Approaches

### Psychological Interventions

The prevalence of full-syndrome BED is relatively low in children and adolescents, and data suggest that subclinical symptoms (e.g., binge-eating episodes that fail to meet size or frequency criteria required for a diagnosis of BED) are strongly associated with distress, impairment, and risk for full-syndrome eating disorders [35, 36]. Thus, interventions for youth have typically relied upon age-adapted criteria for BED, which include both objective and subjective binge episodes and allow for lower frequency and/or limited duration of binge eating relative to DSM-5 criteria [37]. Most youth interventions have focused on developmentally tailoring interventions with demonstrated efficacy in the treatment of adults [38]. These include CBT [39–41], IPT [42], and DBT [43, 44]. CBT has been shown to outperform delayed treatment in youth with recurrent binge eating [41] and age-adapted BED [39], although currently there are no studies comparing it to a credible control treatment. IPT has been found to produce greater improvements in loss of control eating (with or without consumption of an objectively large amount of food) among adolescents than a health education control condition in both a small pilot RCT [45] and a larger, fully powered RCT [42]. Finally, both a case report [44] and a small RCT [43] suggest that a DBT-informed approach may hold promise as an acceptable and efficacious treatment for adolescent binge eating.

### Pharmacological and Medical Interventions

Research on pharmacological agents for the treatment of binge-type eating in youth has been extremely limited, although off-label usage of LDX has been reported in the literature with promising effects on binge-type eating in a single case [46]. Similar to adult studies [23], bariatric surgery in adolescents is associated with improvements in binge-type eating from pre-surgery through up to six years post-surgery [47–49], although prevalence rates tend to show gradual increases with longer passage of time since surgery.

### Limitations and Research Gaps

While CBT, IPT, and DBT all show promise in the treatment of binge eating-related conditions in children and adolescents, few RCTs have been conducted, and even fewer have included a rigorous time- and attention-matched control condition. Therefore, it is premature to declare a “first line” evidence-based approach for this age group. There are several additional limitations and gaps that impede scientific progress in understanding best practices for the treatment of binge eating-related conditions in youth.

First, current treatments for binge-type eating disorders in youth are based on etiological models that were developed for adults, and either have not been adequately tested in youth or have received limited support in youth. For example, restraint theory (on which CBT is based) proposes that rigid and overly restrictive dieting attitudes, cognitions, and behaviors lead to over-reliance on non-physiological cues (e.g., pre-defined portion sizes) to guide eating behavior, increasing risk for dysregulated eating when dietary restraint is interrupted [e.g., through breaking a dietary rule; 50]. Yet, there is mixed support for elevated dietary restraint and objective disturbances in eating behavior in adolescents with binge-type eating [51–61]. Similarly, affect regulation theories (on which IPT is based) suggest that binge-type eating represents an attempt to modulate negative mood [e.g., via affective trade-off or masking; 62]. Yet, while there is strong support for negative affect as a distal risk factor for later development of binge-type eating in adolescents [63, 64], momentary assessment methodologies have yielded no evidence that negative affect is a proximal precipitant of binge-type eating in youth [65–67]. Relatedly, current interventions for youth fail to account for other factors that may independently or interactively contribute to dysregulated eating in youth, such as the food environment and individual responses to palatable foods.

Second, existing interventions fail to adequately consider that self-regulation capacity [i.e., ability to override undesirable impulses in pursuit of longer-term goals; 68]. Self-regulation subsumes the three core executive functions of inhibition, working memory, and cognitive flexibility, and lower order derivative processes including planning, problem-solving, affect regulation, etc. [69]. Executive functions remain in flux throughout childhood, late adolescence, and into early adulthood [70, 71], underscored by developmental changes in neurobiology [72]. This may make it challenging for adolescents to implement skills learned during treatment that largely depend on self-control in the moments and contexts they are needed most (e.g., remembering to employ stimulus control to prevent binge-type in high-risk locations).

Third, as with adult interventions for BED, treatments targeting youth can be costly to administer and may not be adequately covered by insurance [especially those delivered in face-to-face formats; 73], often involve intensive participation (e.g., 16–20 sessions over 6–12 months), typically require extensive training of providers that may not be widely available, and are frequently inaccessible outside of metropolitan areas and specialty treatment settings. Digital interventions may present an alternative to offset some of these limitations, but few have been rigorously tested [40]. Those that have been evaluated utilized outdated technology (e.g., required use of a desktop or laptop computer and were not designed for use via Smartphone or other ambulatory devices) and were not informed by the needs, expectations, and preferences of intended users which may limit engagement and clinical impact.

## Future Directions and Conclusion

Although significant progress has been made over the past several decades in developing and evaluating interventions for BED across the lifespan, there are several major research gaps that should be a focus of research in the coming years. First, although there are several efficacious interventions for BED in adults, with many of these approaches demonstrating efficacy in youth as well, outcomes are heterogeneous and a significant proportion of individuals who receive these interventions fail to respond optimally (e.g., continue to meet criteria for BED or engage in subclinical binge-eating symptoms after a course of treatment). Alternative treatment options for initial non-responders to first-line treatments are not well-characterized, nor are characteristics of individuals who are likely to have poor response to particular treatment modalities. Understanding for whom, and under what conditions, individuals are likely to respond to particular treatment modalities could inform personalized medicine approaches and help with allocation of limited therapeutic resources (e.g., using a lower-touch intervention for individuals likely to respond to a range of approaches while preserving more time- and resource-intensive interventions for those likely to have a more recalcitrant course of illness). Adaptive stepped care interventions and use of sequential multiple assignment randomized trial (SMART) designs will be particularly useful to address these questions and have already shown promise in BED treatment [74•]. Relatedly, mechanisms of action for existing treatments are not well characterized. Identifying the most active ingredients of treatments using multiphase optimization strategies could improve both efficacy and efficiency (e.g., by focusing on only the most potent intervention strategies, thereby reducing the overall length of treatment [75•]). It will also be important to understand how to effectively target such mechanisms in real life. To this end, just-in-time adaptive interventions may assist in personalizing delivery of intervention components to capitalize on the times and places those components will be most impactful [76•].

Next, as mentioned earlier, access to evidence-based treatments is largely limited to those with adequate resources (e.g., insurance coverage, reliable transportation to travel to and from treatment) and/or geographic proximity to specialty centers (which are mostly centered in densely population urban regions). One consequence of the COVID-19 pandemic has been the pivot to telehealth/virtual healthcare for most mental health conditions; however, acceptability and efficacy of telehealth and other virtual approaches (e.g., eHealth/mHealth, digital interventions) are not well characterized.

Finally, identifying adjunctive treatment components that target mechanisms outside of the current theoretical frameworks may help to improve outcomes and address comorbidities that frequently co-occur with BED. For example, given evidence that individuals with BED display diminished neurocognitive performance relative to their peers [77], executive function training has been recommended as a way to improve adherence to psychological interventions [78, 79], particularly those focused on momentary processes that may require high degrees of self-regulation to implement in real-world contexts. While current treatments for BED do not address the role of physical activity, there is also evidence that interventions promoting physical activity could exert effects on binge eating, potentially by improving self-regulation and hedonic hunger. For example, emerging physical activity interventions targeting binge eating in adults were related to reduced binge-eating episodes as well as improvements in depression, anxiety, and food craving [80]. Similarly, understanding ways to optimally integrate weight control strategies into interventions for BED could help streamline treatment for those with comorbid obesity, as weight loss following psychological treatment for BED is typically modest or non-existent [81]. Furthermore, existing evidence has suggested potential of weight management interventions to reduce binge eating in both adult [82] and pediatric populations [83–85], though effects are typically less robust for binge eating outcomes than targeted psychological approaches such as CBT [86–88].

Taken together, there are several efficacious treatment options for those with BED, including self-help, clinician-led psychotherapy, and medical interventions, though the development and evaluation of interventions for youth with BED are more limited compared to adults. However, there remain critical barriers to treatment and gaps in knowledge that underscore the need for continued research, treatment development, and potentially refinement of existing theoretical models. This will likely involve investigation of novel biological, social, and environmental factors that have not been adequately considered in prior work. Moreover, it is imperative that future research consider developmental processes occurring in youth and identify if and how the etiology and maintenance of BED in youth may diverge from adults.

## Data Availability

No datasets were generated or analysed during the current study.
